# Determining the Origin of Deformity in Torsional Femoral Pathology: A Narrative Review and an Illustrative Pilot Study of a Novel Methodology

**DOI:** 10.3390/jcm14186489

**Published:** 2025-09-15

**Authors:** Caterina Chiappe, Alejandro Roselló-Añón, Jorge Más-Estellés, Luis Gil-Santos, Joan Carles Monllau, Vicente Sanchis-Alfonso

**Affiliations:** 1 Department of Surgery, School of Medicine, Universitat Autónoma de Barcelona, 08193 Barcelona, Spain; caterina.chiappe@gmail.com; 2 Department of Orthopedic Surgery, Hospital Arnau de Vilanova, 46015 Valencia, Spain; 3 Center for Biomaterials and Tissue Engineering, Universitat Politècnica de València, 46022 Valencia, Spainlu.gils@telefonica.net (L.G.-S.)

**Keywords:** femoral anteversion, anterior knee pain, derotational osteotomy, CT scan, MRI, 3D technology

## Abstract

**Background:** The Derotational femoral osteotomy (DFO) is an effective surgical treatment for patients with disabling anterior knee pain associated with pathological Femoral anteversion (FAV). However, the complexity in determining the precise origin of the deformity has put limits on its use. This study aims to review the literature to learn how the authors study the origin of the deformity and then provide a new methodology using 3D technology to assess the origin of FAV. **Methods:** A search of the literature was conducted on PubMed utilizing the following search string: “anteversion” and “femur” or “origin” or “CT” or “MRI” or “3D”. In addition, an observational study was conducted on CT scans of six femurs from three female patients with unilateral pathological FAV. This work represents a pilot study and should be considered preliminary. Using the 3DSlicer (version 4.11.20210226), MeshMixer (version3.5), and 3DBuilder software (Microsoft.com), 3D biomodels were generated. A mirrored healthy femur served as a reference. The CloudCompare software (software version 2.13.0) was used to compare volumetric structures and analyze torsional deformities. Torsion at each level was quantified using MATLAB (software version 23.2). **Results:** The 3D technology identified three torsional patterns: 1. FAV predominantly originating at the femoral head (distance between the centroids = maximum deformity in the last discs, which coincides with the proximal region of the femur; heat maps = red in the proximal femur); 2. FAV primarily affects the mid-distal diaphysis (distance between the centroids = maximum deformity in the first discs, which coincides with mid-distal third of the femur; heat maps = red in the diaphyseal level); 3. a pan-diaphyseal deformity involving the entire femur (distance between the centroids = both the first and last discs, means deformity along the entire femur; heat maps = red along the entire femoral diaphysis). **Conclusions:** All femoral segments contributed to the total FAV, but the location and severity varied among the cases. Pathological FAV is a multifactorial deformity that can arise in different femoral regions. Individualized correction strategies are essential to improving DFO outcomes and preventing secondary deformities. It is important to note that the pilot data is intended to be purely illustrative and, as such, should not be utilized for the purposes of guiding clinical decision-making.

## 1. Introduction

The Derotational femoral osteotomy (DFO) is a surgical procedure that produces good clinical results in patients with disabling anterior knee pain (AKP) and increased Femoral anteversion (FAV) unresponsive to conservative treatment [1,2,3,4]. The complexity in the indication for DFO lies both in determining the magnitude of correction required and in identifying the precise origin of the deformity. Although FAV has been extensively studied, only recently has an attempt been made to localize its point of origin [5,6,7,8]. There is controversy in the literature as to the best method for determining the origin of the deformity. As demonstrated in a previous work [9], both the distal and proximal segments of the femoral diaphysis contribute to torsional deformity in FAV, which is a pan-diaphyseal disorder.

Determining the origin of the femoral torsion is recommended, as any osteotomy should be performed at the level of the deformity. Performing an osteotomy anywhere other than the origin of the deformity corrects the malformation, but a new deformity of the same magnitude and in the opposite direction could be generated. Different methods for measuring the origin of the deformity are found in the literature. Some methods are based on CT images [5,6,7,8,10] and others on MR images [11,12,13,14,15,16,17]. Some studies employ 3D technology in orthopedic surgery and traumatology [18,19,20,21,22,23] and use a mirror image of the healthy side to establish the physiological reference of the patient [24,25]. The objective of this study is to investigate and extend our understanding of the origin of femoral anteversion. For this reason, a narrative review of the literature was initially conducted to serve as a prologue to the presentation of a new methodology. The new methodology is based on the use of 3D technology to assess the origin of FAV. To achieve this, various software programs are utilized to compare similarities between volumetric structures [26,27].

The hypothesis of our work is that the origin of the deformity is variable in each femur and depends on both the neck of the femur as well as the entire femoral diaphysis.

## 2. Narrative Review

**Figure 1:**
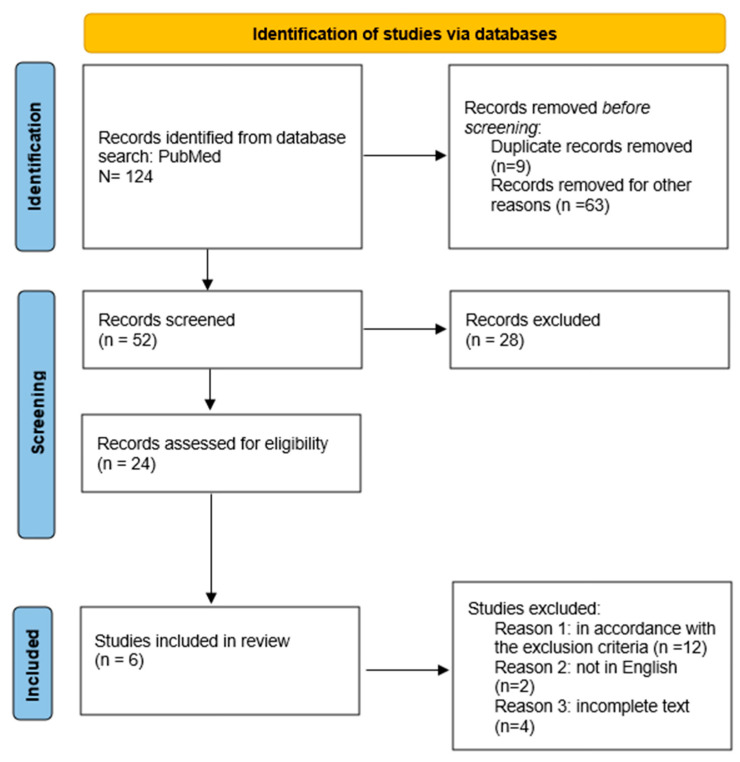
PRISMA flow diagram.

The first branch of this study is the narrative review of the literature. Literature research was performed on PubMed utilizing the following search string: (“femoral anteversion” OR “femoral version” OR “femoral internal rotation” OR “hip anteversion”) AND (“origin” OR “physiopathology” OR “ Anatomy”) NOT (“arthroplasty”). The following criteria were applied in the compilation of the relevant studies. (Figure 1). Firstly, the origin of the anteversion was analyzed, and the contribution of femoral segments was then considered. Only studies that satisfied these criteria were included. Studies that exclusively analyzed anteversion measurements were excluded from the present analysis (Table 1 and Table 2).

Afterwards, a retrospective observational study on the CT scans of patients with unilateral pathological FAV was conducted.

### 2.1. Anatomic Method

In 2019, Archibald et al. [5] analyzed 590 bilateral pairs of cadaver femurs (1180 femurs in total) to gain a clearer understanding of where rotational deformities occur. A random selection was made from the Hamann–Todd Osteological Collection (Cleveland, OH, USA), which comprises over 3000 disarticulated human skeletons. These were systematically catalogued by age, sex, and ancestry within an extensive database. Their extensive cadaver-based research examined torsional variation both above and below the lesser trochanter. They defined the total femoral version as the angle on the axial plane between the femoral neck and the posterior condyles of the femur. Shaft torsion was described as the axial angle between the lesser trochanter and the posterior femoral condyles. The version of the femoral neck was calculated by subtracting the shaft torsion from the total femoral version. That was the first study to assess how strongly each part of the femoral version correlates with the overall femoral version. Their findings indicated that both the neck and the shaft influence femoral torsion, though the neck version showed a slightly stronger association. However, it should be noted that neither the neck version nor the shaft torsion provides a complete predictive model for the overall femoral version.

### 2.2. Methods Employing CT

Computed tomography (CT) is widely regarded as the gold standard for the assessment and quantification of lower limb torsional deformities, owing to its cost-efficiency, rapid image acquisition, and high accuracy in delineating osseous landmarks within the axial plane [28].

Kim et al. [6] studied a group of young patients (average age 14 years, ranging from 3 to 28 years) who exhibited abnormal gait characterized by inwardly rotated feet due to medial femoral torsion. A total of 41 patients were assigned to the intervention group, with 20 patients in the cerebral palsy (CP) subgroup and 21 patients in the developmental medial femoral torsion (DF) subgroup. The control group consisted of 26 patients. They assessed FAV using the method introduced by Rippstein [29]. It defines FAV as the angle between the femoral neck axis and the posterior tangent line connecting the medial and lateral femoral condyles. To evaluate infratrochanteric torsion (ITT) and supratrochanteric torsion (STT), they identified the intertrochanteric line (ITL), which runs along the axis between the lesser and greater trochanters. STT was defined as the angle between the ITL and the femoral neck axis, while ITT was the angle between the ITL and the condylar axis. The study showed that FAV abnormalities can occur in the supratrochanteric region as well as the infratrochanteric region. STT decreased during development, leading to reduced global FAV in the DF subgroup, whereas a decrease in STT was counterbalanced by an increase in ITT in the CP subgroup, leaving the global FAV unchanged. Consequently, corrective procedures such as derotational osteotomy should target the specific site of deformity to avoid the development of secondary lever arm issues.

Waisbrod et al. [8] proposed, in a cohort of patients with gait abnormalities, hip pain, and patellar instability, that FAV can be considered a subtrochanteric deformity. They assessed femoral torsion (FT) and lesser trochanter torsion (LTT). FT was determined using Murphy’s method, which involves measuring the angle between the femoral neck axis and the posterior bicondylar axis, where positive values indicate anteversion and negative values indicate retroversion. LTT reflects the rotational alignment of the lesser trochanter relative to the femoral shaft and was measured using Herzberg’s method. This involved identifying an angle between a line bisecting the lesser trochanter at its thickest midpoint and a baseline through the femoral condyles [30]. They then calculated the combined angle (CoA) as the difference between FT and LTT. The CoA, representing the angle between the femoral neck axis and the LTT, reflects supratrochanteric torsion, while LTT indicates torsion at the intertrochanteric level. By analyzing both angles in relation to the overall FT, they were able to pinpoint the precise location of the torsional abnormality, whether it lies above or below the lesser trochanter. Their findings showed a linear relationship between both LTT and CoA with FT. However, LTT increased at a rate three times greater than CoA, suggesting that as FT increases, rotational deformity develops both above and below the lesser trochanter, but is significantly more pronounced in the subtrochanteric region. The authors emphasized the importance of addressing the deformity at its origin through targeted osteotomy to ensure effective correction and avoid muscle imbalance. Their results support subtrochanteric derotational osteotomy as the most appropriate surgical intervention.

In a previous study, Sanchis-Alfonso V et al. [9] conducted a segmental analysis of femoral torsion by dividing it into two parts using Murphy’s method: proximal femoral torsion (above the lesser trochanter) and distal femoral torsion (below the lesser trochanter). Their findings indicated that abnormal FAV results from an increased anteversion at the femoral neck in patients with AKP and is combined with a compensatory decrease in torsion within the femoral shaft that is oriented in the opposite direction. Rather than being attributed solely to the neck or diaphysis, total FAV reflects a global alteration affecting the entire femur. Furthermore, they discovered a strong inverse relationship between neck torsion and opposite-direction diaphyseal torsion, implying that the diaphysis attempts to offset the excessive torsion of the neck. However, this compensatory mechanism breaks down in pathological cases. Performing an osteotomy at the level of the lesser trochanter may help address both deformities simultaneously. This finding supports the use of an intertrochanteric osteotomy as a viable treatment option for FAV in female patients suffering from AKP [9].

### 2.3. Methods Employing MRI

In the literature, there are several methods for measuring AVF with MRI [11,12,13,14,15,16,17], but only one study has used MRI to determine the origin of anteversion and the contribution of the femoral segments.

Seitlinger et al. [7] assessed femoral torsion at various anatomical levels to understand each level’s contribution to overall femoral torsion and to examine the torsion distribution pattern in patients with pathological torsion compared to a control group. To measure FAV, they drew four lines: the first through the center of the femoral head and neck (FN); the second from the midpoint of the lesser trochanter (LT) to the center of the femoral shaft, defined as the midpoint of a circle fitting the shaft; the third tangent to the posterior edge of the distal femur (DF); and the fourth tangent to the posterior condyles (PCs). They then calculated angles between these lines: FN to PCs (total femoral torsion), FN to LT (neck torsion), LT to DF (mid-shaft torsion), DF to PCs (distal torsion), and LT to PCs (shaft torsion).

Their findings showed that both the femoral neck and diaphysis contribute to femoral torsion in patients with patellar instability and increased FAV. They also observed a negative correlation between neck and shaft torsion, indicating a compensatory mechanism, where increased internal torsion at the neck is generally balanced by external torsion of the shaft. When overall torsion exceeds 25°, there is a marked rise in internal neck torsion accompanied by an even greater reduction in external torsion in the diaphysis, resulting in a significant increase in total femoral torsion.

However, MRI, while effective, is a lengthy and costly procedure that may be complicated by metal implants, patient positioning issues, and variability in measurements [31,32].

### 2.4. Methods Employing 3D Technology

Joan Ferrás-Tarragó et al. [27] utilized 3D technology to analyze the femoral structure and gain deeper insight into femoral torsion patterns. They created a 3D biomodel of a femur from a patient with unilateral pathological FAV. By generating a mirror image of the unaffected side, they superimposed it onto the symptomatic femur for comparison. The establishment of the triangular system on the same horizontal plane was achieved by employing two reference points, one distally and one proximally. The femurs were measured to determine the morphological discrepancy in their longitudinal axis. This was achieved by taking measurements in millimeters between the two femurs in three zones: the femoral neck, the proximal diaphysis, and the distal diaphysis (Hausdorff–Besicovitch method). Consequently, an elevated disparity in millimeters at a particular measuring point between the femurs is indicative of diminished anatomical similarity. Their results indicated that femoral torsion originates primarily in the supracondylar region, with the rotational axis aligned along the length of the femoral shaft. The study revealed a trimodal pattern of deformity: one deformity located in the femoral shaft near the supracondylar area, and two additional, more pronounced deformities at the level of the proximal femur, specifically involving the neck and femoral head.

## 3. Methods

The secondary branch of this study was to conduct a pilot study utilizing new 3D technology to assess the origin of FAV in a limited patient cohort. To achieve this, various software programs are utilized to compare similarities between volumetric structures. All the software used is completely free and compatible with most computers on the market.

A retrospective observational study was performed on the CT scans of patients with unilateral pathological FAV. Six femurs were included from three patients with AKP and unilateral pathological FAV who, due to specific circumstances, required a CT scan of the entire femur for surgical planning. All the patients were female and ranged in age from 18 to 30 years. FAV values greater than 15° were considered pathological. The exclusion criteria were a history of femoral trauma (fractures) or previous surgery. Given the limited sample size, which was due to the low prevalence of unilateral femoral torsional deformities, this study was considered a pilot study.

First, CT images of both femurs were downloaded in the DICOM (Digital Imaging and Communications on Medicine) format, and the 3D biomodel of both femurs was obtained with the 3D Slicer software (^®^Harvard Medical School, Boston, MA, USA).

**Figure 2:**
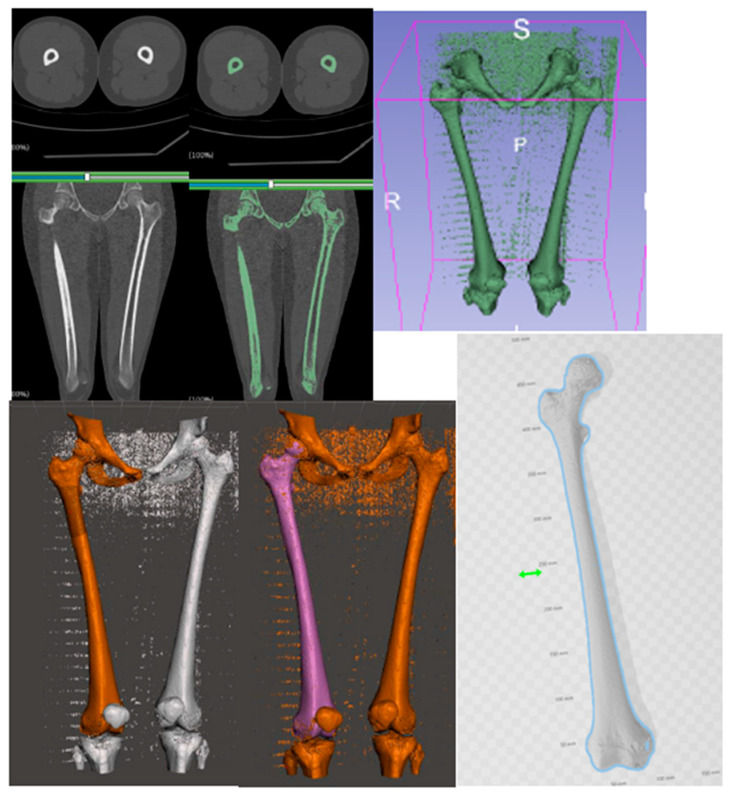
The process to obtain a clean femur biomodel. Software: 3D Slicer, MeshMixer, 3D Builder.

Subsequently, elements other than the femur (pelvis, soft parts, patella, and tibia) were extracted using MeshMixer (Autodesk Inc.^®,^ Mill Valley, CA, USA) to create a clean biomodel (Figure 2).

**Figure 3:**
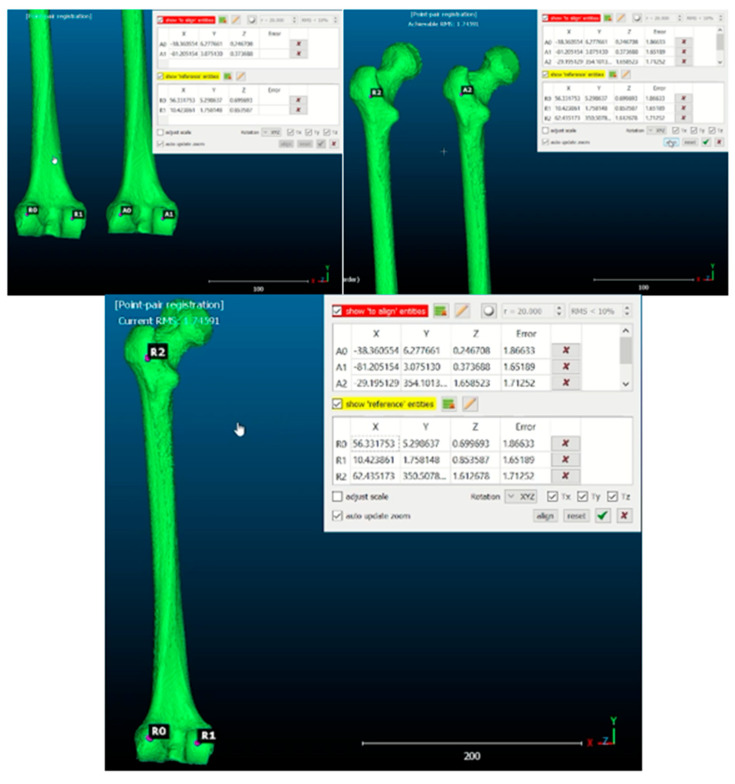
Cloud Compare software: The mirror image of the healthy side is obtained, and landmarks are set on the femoral condyles and proximal femur to align the healthy femur with the pathological femur.

Afterwards, the specular image of the healthy side was made using the 3D Builder software (Microsoft Corporation^®^, Washington, WA, USA), and both femurs were aligned (healthy specular and pathological), taking the horizontal as the neutral reference plane and marking the contact points of the femoral condyles and the greater trochanter with the horizontal. This was carried out to establish easily reproducible reference points for the alignment of both femurs (Figure 3, Video S1).

To analyze the difference in the deformity between the healthy (specular) and pathological biomodels of each patient, the CloudCompare^®^ (software version 2.13.0; open-source software; www.cloudcompare.org (accessed on 5 May 2025) was used. This is a program that generates a three-dimensional mesh of points of the entire surface of the femur, in which each point corresponds to a point in the XYZ space coordinate system.

**Figure 4:**
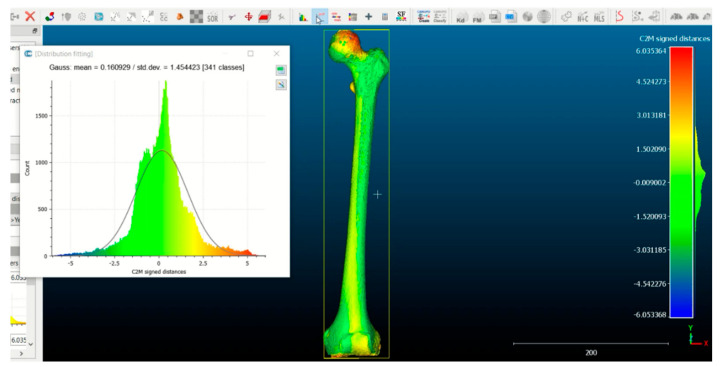
Cloud Compare software: Mesh of three-dimensional points and expression of the distance between points through a heat map.

Two points were fixed on the distal femoral condyles. The connection of those points defines the X reference axis. A point was then fixed at the proximal end of the femur, and the union of that point with one of the first two defines the Y reference axis (longitudinal axis of the femur). Once these two axes are fixed, the axis perpendicular to them at their point of intersection that makes up a dextrorotatory reference system defines the Z reference axis (Figure 4).

The data was recorded in an Excel spreadsheet. The calculation procedure involved dividing each femur into multiple discs (N = 20), perpendicular to the Y-axis. The spreadsheet was utilized to determine the initial and final points of each femur. By calculating the difference between these points (the femur length) and dividing by N, the thickness of each disc was determined, and shown to be of equal thickness. The coordinates of the centroid or geometric center of all the points of that disk (XG1, YG1, ZG1) were also calculated.

### 3.1. Calculation of Femur Centroid Position

The spreadsheet was employed to denote all points that belong to the first disk (the distal position), and to calculate the coordinates of the centroid, otherwise known as the geometric center, of all points on that disc. The centroid’s coordinates are specified as (X_G1_, Y_G1_, Z_G1_). For the *Y_G_*_1_ coordinate, the midpoint of the disc is taken. Assuming that *Y_P_*_1_ denotes the *Y*-coordinate of the first point of disk 1, and *Y_U_*_1_ denotes the *Y*-coordinate of the last point of disk 1, then the following equation is true:

$$Y_{G1}=Y_{P1}+\frac{Y_{U1}-Y_{P1}}{2}$$

The remaining two coordinates are obtained by adding the respective coordinates of all the points of that disc and dividing the result by the number of points of that disc (*N*_1_).

$$X_{G1}=\frac{\sum_{1}^{N_{1}}x}{N_{1}}\;\;\;\;\;Z_{G1}=\frac{\sum_{1}^{N_{1}}z}{N_{1}}$$

**Figure 5:**
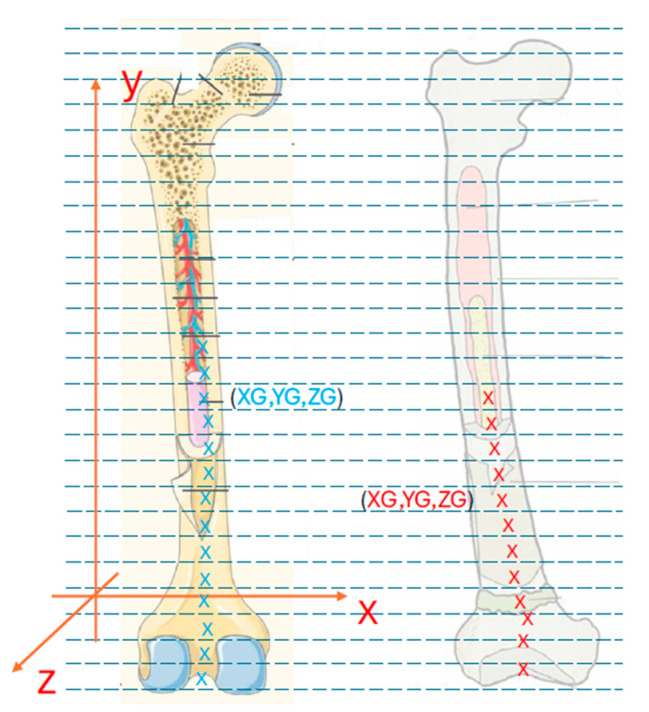
1—Segmentation of both femurs into N = 20 discs. 2—Determination of the centroid position. 3—Superposition of one end of the femurs, and then calculation of the distances between the centroids of the corresponding discs of both femurs (discs with the same YG coordinate), in the X and Z directions (XG and ZG). 4—Graphical representation of the distance between the centroids as a function of the YG coordinate of the centroid, which allows evaluation of the deformation of the pathological femur.

The distance between the centroids in the *X* and *Z* directions is expressed as *X_G_* and *Z_G_* (Figure 5).

### 3.2. Calculation of the Femur Rotation in the XZ Plane

**Figure 6:**
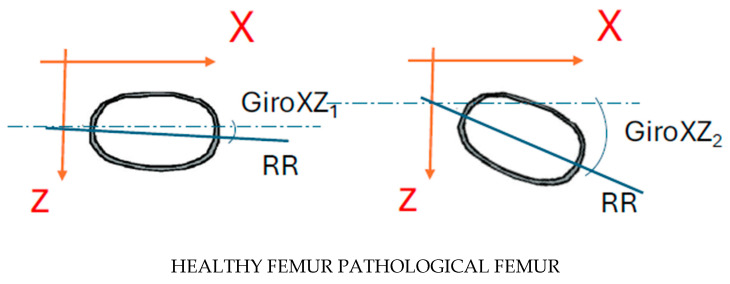
The difference GiroXZ1–GiroXZ2 indicates the torsion suffered by a section of the pathological femur with respect to the healthy one.

The regression line of all the points of each disc on the *XZ* plane was calculated, which indicates the rotation suffered by the femur around the *Y* axis in each disc (Figure 6).

z=mx+b

The parameter m is representative of the trigonometric tangent of the angle that the regression line forms with the *X* axis. This can be calculated by determining the inverse tangent function.

$$\alpha =\tan^{-1}a$$

The alterations in the angle values for the various discs of the femur signify the rotation undergone by the femur around the Y-axis in each disc, thus providing an approximate conception of the alterations in the orientation of the discs along the femur, and the disparities between the healthy and the pathological femur.

To replicate the processes in each of the discs, for each of the femurs (healthy and pathological), the Matlab program (modelofemurv5.m) was utilized.

## 4. Results

Analyzing the data from the three femurs, both the proximal and distal femoral segments were seen to contribute to the total FAV, albeit with a different pattern in the three cases. The results that follow are based on a pilot study that used new 3D technology to assess the origin of FAV. Therefore, they are merely illustrative.

**Figure 7:**
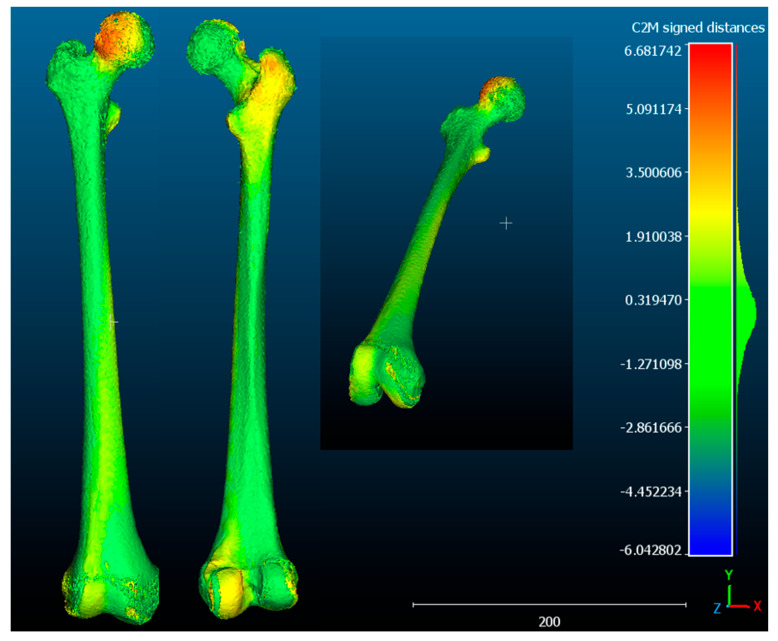
**Cloud Compare**: The difference in the distance between the points is illustrated by a heat map of the pathological femur. One can see where the highest percentage of deformity is concentrated (red indicating maximum deformity, green in the absence of deformity, blue showing deformity in the opposite direction (negative). In this case, the percentage of deformity is higher at the level of the proximal femur. The results are merely illustrative.

**Figure 8:**
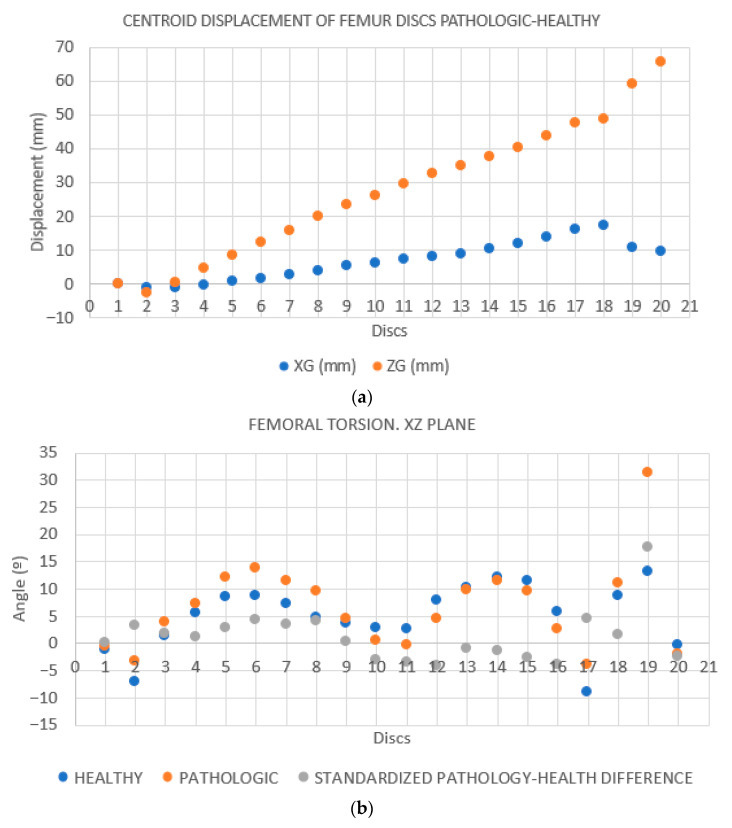
(**a**): **Distance between the centroids according to the YG-coordinate of the centroid.** Each point on the plot corresponds to one disk (N = 20 disks). The first disc, at the level of the femoral condyles, corresponds to point 1. The distance between the centroids of each disc in the X and Z directions is expressed as XG and ZG, and allows for the evaluation of the deformity of the pathological femur. In this case, the maximum deformity of the pathological femur is seen in the last discs, which coincides with the proximal region of the femur. The results are merely illustrative. (**b**): **Calculation of the regression line of all the points on the disks on the XZ plane. Rotation of the femur around the Y-axis on each disk**. The difference between the angles of the healthy and pathological femur corresponds to the torsion of the pathological femur with respect to the healthy femur (gray dots). In this case, there is a slight increase in torsion in the first discs (discs 1–8 distal femur), a successive decrease in torsion, and finally a greater increase in the last discs (discs 17–19 proximal femur and femoral head). The results are merely illustrative.

**Case 1**. The FAV is predominantly of proximal origin, and the femoral head is where the highest percentage of torsion is achieved. This can be seen in the heat map from the CloudCompare program (Figure 7), where the red coloration is concentrated in the femoral head. The intertrochanteric region shows an orange coloration, and both the diaphysis and the external femoral condyle are shown in yellow. This implies that there is a lower degree of torsion in these latter regions, but not the absence of deformity. These findings are supported by the data exported from Excel. Then, the data was analyzed and is reported in Figure 8a,b.

**Figure 9:**
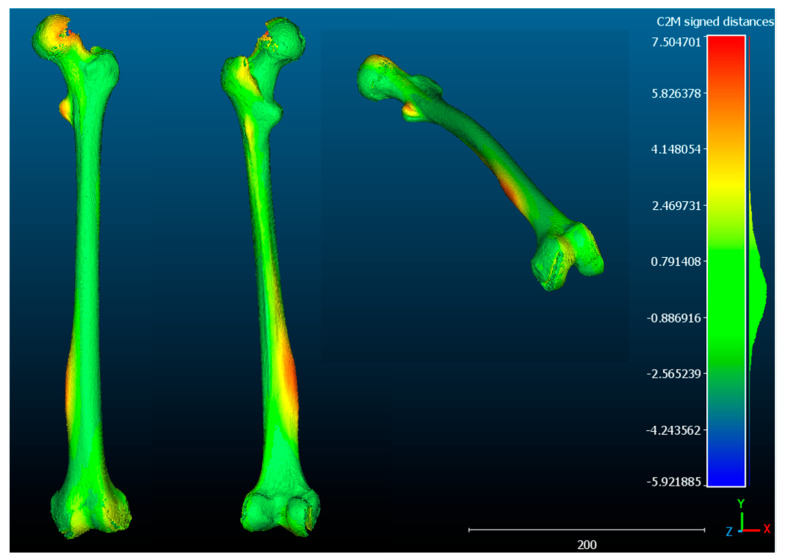
**CloudCompare**: The highest percentage of deformity (red) is found at the diaphyseal level, in the mid-distal third. In the lesser trochanter and femoral head, there is a minor degree of deformity (orange) and a slight torsion at the diaphyseal level (yellow). The results are merely illustrative.

**Figure 10:**
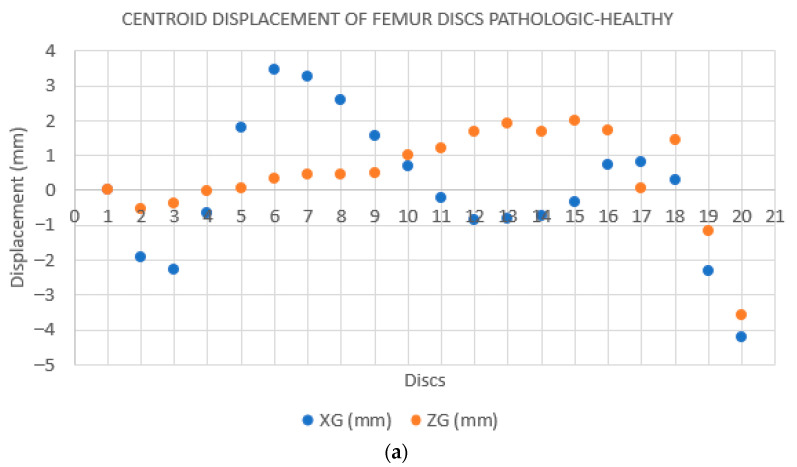

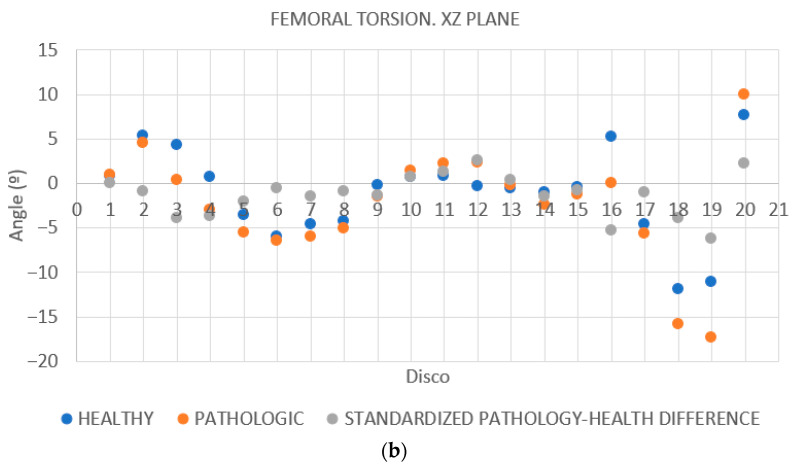
(**a**): **Distance between the centroids based on the YG-coordinate of the centroid.** The greatest distance is observed in the first discs (4 to 9), coinciding with the mid-distal third of the femur. Subsequently, another significant increase is seen in the proximal femur (discs 12–15). The results are merely illustrative. (**b**): **Calculation of the regression line of all the points of the disks on the XZ plane. Rotation of the femur around the Y-axis on each disk.** The greatest torsion is shown in discs 5 to 12, matching the mid-distal third of the femur. In addition, another peak is seen in the proximal portion of the femur (discs 17–20). The results are merely illustrative.

**Case 2.** The FAV is predominantly of distal origin, with the highest percentage of torsion found in the mid-distal third of the diaphysis. The heat map from the CloudCompare program (Figure 9) and the graphs (Figure 10a,b) show the twisting pattern.

**Figure 11:**
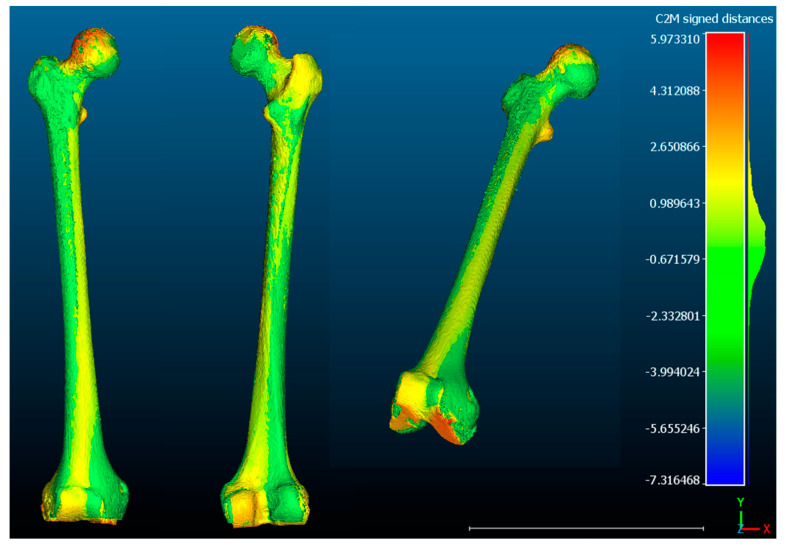
**CloudCompare**: An increase in deformity is evident along the entire femoral diaphysis (orange–yellow), with a higher percentage of deformity in both the femoral head and femoral condyles (red–orange color). The results are merely illustrative.

**Figure 12:**
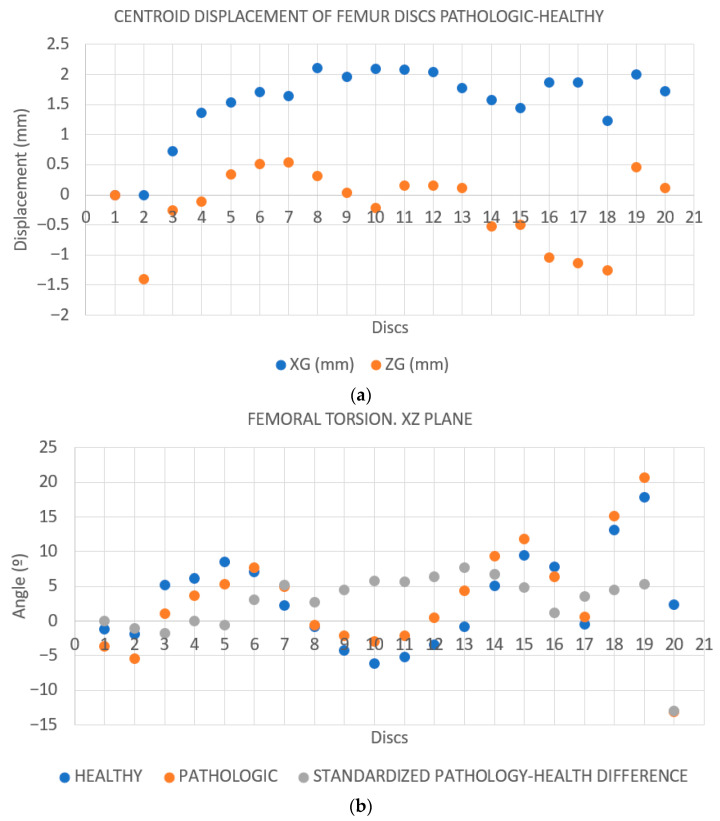
(**a**): **Distance between the centroids based on the YG-coordinate of the centroid.** The increase in the distance between the discs is evident along the entire femur, being more marked in the first discs (discs 1–2 femoral condyles) and in the last discs (discs 16–20 trochanter and femoral head). The results are merely illustrative. (**b**): **Calculation of the regression line of all the points on the disks on the XZ plane. Rotation of the femur around the Y-axis on each disk**. A torsion pattern distributed along the entire length of the femur is observed. The results are merely illustrative.

**Case 3.** Both the distal and proximal regions contribute to the development of FAV. In addition, the rotation is present along the entirety of the femoral diaphysis as shown in Figure 11 and Figure 12a,b.

## 5. Discussion

Our study has highlighted two important findings. The first one is that, of all the methods described, the 3D method appears to be the most promising. The second one is that all the femoral segments contribute to the total FAV.

As demonstrated in extant research, a variety of methods have been described in the medical literature. They include anatomical methods, those utilizing CT, MRI-based methods, and, more recently, 3D-based methods.

The anatomical method presented by Archibald, the investigation being primarily osteological and radiological in nature, did not incorporate key clinical factors such as muscle insertion sites and their functional implications. This limits the ability to fully understand the clinical relevance of the observed anatomical deformities.

For this purpose, subsequent studies were performed on CT images in clinical subjects.

The 2012 paper by Kim was the first to analyze the origin of the deformity using CT, but there are further limitations posed by the study population itself. The cohort predominantly included patients with medial torsion deformities. In contrast to the control and developmental medial femoral torsion groups, these findings are not readily generalizable to the typical clinical progression seen in pediatric cerebral palsy populations. Furthermore, the methodology employed to ascertain the origin of the deformity may result in variations in the measurements obtained. This is because the use of the intertrochanteric line (ITL) as an anatomical reference axis was not fully standardized, and the criteria for selecting CT slices to align the lesser and greater trochanters were not clearly described. These factors have the potential to impact measurement consistency.

Subsequently, Waisbrod conducted another study with the same aim in 2017. The main limitation of this study is that it is radiological rather than clinical, meaning that the functional and symptomatic implications of the deformities were not evaluated. Additionally, the sample comprised a pre-selected group referred to CT scans based on varying clinical indications, which limits the applicability of the results to asymptomatic populations due to potential selection bias.

In 2023, the Sanchis’ group placed significant importance on the correlation between the correct measurement of the deformity, the site of the corrective osteotomy, and the clinical results; but again, the sample population is limited to symptomatic patients. The findings specifically pertain to young female patients presenting with AKP, restricting generalizability because some individuals with FAV exhibit gait abnormalities without AKP. Indeed, the classification of groups based on clinically observable FAV assessed by a single evaluator introduces an additional source of variability.

### 5.1. Limitations Related to Imaging Modalities Also Require Consideration

The use of CT exposes patients—particularly younger individuals—to ionizing radiation, which poses ethical and safety concerns. Consequently, non-radiative modalities such as MRI have been adopted in some contexts to study deformity origins.

Only one study has used MRI to determine the origin of anteversion and the contribution of the femoral segments. The work of Seitlinger in 2017 analyzed the contribution of femoral segments to the FAV, but the absence of clearly defined threshold values for categorizing torsion as high or low in clinical practice complicates interpretation and the application of the results.

MRI does have its own drawbacks. They include prolonged acquisition times, higher costs, sensitivity to metal implants, patient positioning challenges, and measurement variability [31,32].

Finally, anatomical variability, particularly regarding the greater and lesser trochanters, limits the reliability of traditional two-dimensional (2D) landmarks in assessing femoral torsion. The assumption that the lesser trochanter occupies a fixed position on the proximal femur is questionable and may lead to the misinterpretation of torsional origins when using linear models. This variability highlights the need for more precise methodologies, such as volumetric reconstruction and three-dimensional superposition techniques, which provide enhanced spatial accuracy for localizing torsional deformities.

In comparison with conventional 2D methodologies, 3D measurement techniques have been shown to possess enhanced reliability for the assessment of segmental femoral torsion. This enhanced consistency can be attributed to the inherent benefits of three-dimensional imaging. Specifically, 3D techniques facilitate precise reconstruction of the intricate 3D anatomy of the femur, thereby circumventing the inherent limitations of 2D approaches like the superimposition of anatomical structures. In addition, CT-based 3D reconstructions facilitate the precise identification of osseous landmarks, thereby reducing the risk of inaccuracies in measurements associated with soft tissue overlap or the indistinct anatomical references in 2D imaging [33].

Some studies employ 3D technology in orthopedic surgery and traumatology, using a mirror image of the healthy side to establish the physiological reference of the patient [18,19,20,21,22,23,24,25]. The latter is the morphological target to be achieved with corrective osteotomy.

Based on the findings of those studies, Ferras et al. [27] employed a new methodology to assess the origin of the deformity in 2020. The employment of mirror imaging of the healthy side facilitates surgical correction aimed at restoring the anatomical symmetry of patients. This methodology involved the utilization of 3D technology and various software programs to compare similarities between volumetric structures. The main limitation of this innovative study is its technical aspect, specifically the complexity of the software. Another limitation is its restricted commercial availability, given its pay-per-use model.

The capacity of 3D measurements to faithfully represent lower limb torsional profiles facilitates more precise preoperative planning, particularly in determining the indication for derotational osteotomies and in optimizing the correction required [33].

Our study demonstrates that pathological FAV depends on all the femoral segments and is variable in each femur. In the initial case, the etiology of the deformity is predominantly located at the proximal level, exhibiting a substantial increase in torsion at the femoral head. The second case, on the other hand, demonstrates a predominance of deformity in the mid-distal third of the diaphysis. Finally, the third case demonstrates a torsional pattern distributed along the entire length of the femur, emphasizing the complexity of the phenomenon and the need for an individualized approach for each patient [5,6,7,8,9]. While some advanced imaging programs require costly licenses and specialized hardware, our study utilizes freely available software compatible with standard computing platforms, which aids in faithful reproducibility.

### 5.2. Limitations of Our Method

The main limitation is the small sample size, which is due to the low prevalence of unilateral femoral torsional deformities. Therefore, it is not the intention of this pilot study to influence the decision-making processes of orthopedic surgeons in their clinical practice. This pilot study yielded several preliminary findings that indicate the need to expand the sample size and continue the research. This only represents the initial step in the long process of validating a new measurement technique.

Although three-dimensional biomodels provide an accurate representation of the femoral anatomy, the interpretation of the results may be influenced by the process of image superimposition and alignment. Moreover, the implementation of 3D technology in routine clinical practice could lead to technical limitations due to the learning curve associated with the use of 3D software. Conversely, the software utilized is entirely free and compatible with most commercially available computers, thus circumventing resource restrictions.

### 5.3. Clinical Relevance of Our Method

It is important to study the origin of a deformity because a correction at a level different from the maximum deformity may have undesirable effects on the patient. Performing osteotomies at different levels would have similar effects on overall torsion, correcting the degrees of total FAV. However, this could lead to unbalanced over-rotation at one level and under-rotation at another. Even if the total pathological FAV angle is corrected, a new deformity of the same magnitude and in the opposite direction could be generated, or a new deformity plane could be created on the coronal or sagittal [7,26,27].

## 6. Conclusions

The identification of the origin of deformities in FAV poses significant challenges to orthopedic surgeons. The optimal method remains to be determined. This study preliminarily demonstrates the usefulness of 3D technology to identify the origin of deformity in pathological femoral anteversion and highlights the importance of a personalized approach to surgical planning. These findings could contribute to optimizing the results of Femoral derotational osteotomy and reducing the incidence of secondary deformities. Nonetheless, further studies with a larger number of participants are required to provide more robust evidence on this matter.

## Figures and Tables

**Table 1:** The search strategy.

Items	Specification
Date of search	10 June 2025
Databases and other sources searched PubMed	PubMed
Search terms used	“Origin”—“Femoral anteversion”
Timeframe	1995–2025
Inclusion criteria	Studies analyzing the origin of the anteversion and the contribution of femoral segments
Exclusion criteria	Studies exclusively analyzing anteversion measurements

**Table 2:** Methods employed.

Method	Year and Authors	Description	Conclusion
ANATOMIC	Archibald et al. (2019) [5]	Total femoral version: the angle between the femoral neck and the posterior condyles. Shaft torsion: angle between the lesser trochanter and the posterior condyles. The version of the femoral neck was calculated by subtracting the shaft torsion from the total femoral version.	Both the neck and the shaft influence femoral torsion. The neck version showed a stronger association.
CT	Kim et al. (2012) [6]	They identified the intertrochanteric line (ITL), which runs along the axis between the lesser and greater trochanters, to evaluate infratrochanteric torsion (ITT) and supratrochanteric torsion (STT).	FAV could arise in the supratrochanteric region, infratrochanteric region, or both.
CT	Waisbrod et al. (2017) [8]	Measured femoral torsion (FT) and lesser trochanter torsion (LTT). They calculated the combined angle (CoA) as the difference between FT and LTT.	FAV can be considered a subtrochanteric deformity.
CT	Sanchis-Alfonso V. et al. (2023) [9]	Segmental analysis of femoral torsion by dividing femoral torsion into 2 segments: proximal femoral torsion (above the lesser trochanter) and distal femoral torsion (below the lesser trochanter).	Strong inverse relationship between neck torsion and opposite-direction diaphyseal. This compensatory mechanism breaks down.
MRI	Seitlinger et al. (2016) [7]	Measured femoral torsion at 4 different levels: neck torsion, mid torsion, distal torsion, shaft torsion, and total femoral torsion	Both the femoral neck and diaphysis contribute to femoral torsion. Negative correlation between neck and shaft torsion, indicating a compensatory mechanism.
3D	Joan Ferrás-Tarragó et al. (2020) [27]	3D biomodel of a femur. Obtained the mirror image of the healthy side and overlapped it with the pathological femur. Evaluated morphological differences between the two femurs across three regions: the femoral neck, the proximal shaft, and the distal shaft.	Femoral maltorsion originates in the supracondylar area, and its rotational axis is the longitudinal axis of the femoral diaphysis.

## Data Availability

The original contributions presented in this study are included in the article/Supplementary Materials. Further inquiries can be directed to the corresponding author(s).

## Supplementary Materials

The following supporting information can be downloaded at: https://www.mdpi.com/article/10.3390/jcm14186489/s1. Video S1: alignment protocol.
